# GPCR-BERT: Interpreting Sequential Design of G Protein-Coupled Receptors Using Protein Language Models

**DOI:** 10.1021/acs.jcim.3c01706

**Published:** 2024-02-10

**Authors:** Seongwon Kim, Parisa Mollaei, Akshay Antony, Rishikesh Magar, Amir Barati Farimani

**Affiliations:** † Department of Chemical Engineering, Carnegie Mellon University, Pittsburgh, Pennsylvania 15213, United States; ‡ Department of Mechanical Engineering, Carnegie Mellon University, Pittsburgh, Pennsylvania 15213, United States; § Department of Biomedical Engineering, Carnegie Mellon University, Pittsburgh, Pennsylvania 15213, United States; ∥ Machine Learning Department, Carnegie Mellon University, Pittsburgh, Pennsylvania 15213, United States

## Abstract

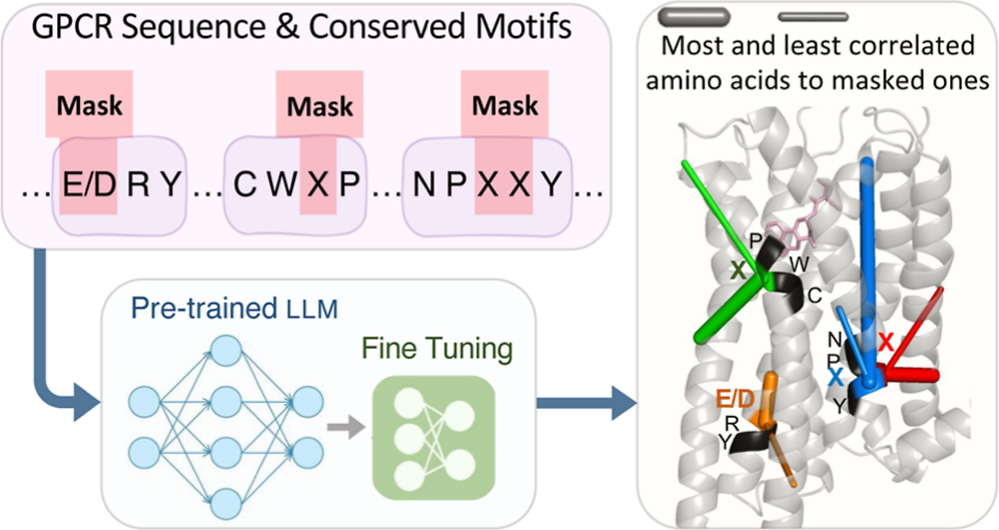

With the rise of transformers and large language models (LLMs) in chemistry and biology, new avenues for the design and understanding of therapeutics have been opened up to the scientific community. Protein sequences can be modeled as language and can take advantage of recent advances in LLMs, specifically with the abundance of our access to the protein sequence data sets. In this letter, we developed the GPCR-BERT model for understanding the sequential design of G protein-coupled receptors (GPCRs). GPCRs are the target of over one-third of Food and Drug Administration-approved pharmaceuticals. However, there is a lack of comprehensive understanding regarding the relationship among amino acid sequence, ligand selectivity, and conformational motifs (such as NPxxY, CWxP, and E/DRY). By utilizing the pretrained protein model (Prot-Bert) and fine-tuning with prediction tasks of variations in the motifs, we were able to shed light on several relationships between residues in the binding pocket and some of the conserved motifs. To achieve this, we took advantage of attention weights and hidden states of the model that are interpreted to extract the extent of contributions of amino acids in dictating the type of masked ones. The fine-tuned models demonstrated high accuracy in predicting hidden residues within the motifs. In addition, the analysis of embedding was performed over 3D structures to elucidate the higher-order interactions within the conformations of the receptors.

## Introduction

Understanding the fundamental function of proteins, as the most important molecules in life, enables the development of improved therapeutics for diseases. Proteins are composed of amino acids arranged in particular orders (sequences) that define the structures and functions of living organisms.^1–5^ The protein sequence, structure, and function are the results of millions of years of evolution and refinement driven by the survival needs of living cells.^6–8^ One of the most fundamental questions in biology is how thousands of proteins have evolved with versatile functions with only 20 different types of amino acids as their building blocks.^9–11^ While significant progress has been made in identifying protein structures and functions through experimental methods, bioinformatics approaches provide valuable insights by leveraging computational tools and algorithms.^12–15^ For example, bioinformatics protein sequence models take advantage of coevolution and a vast amount of proteomics and genomics data to enable prediction of structure or function. However, deciphering the complex relationships between elements of sequences (amino acids) and their functions still remains a challenge as bioinformatics models are not sophisticated enough to model higher-order interactions within amino acid sequences. In recent years, artificial intelligence (AI) has made significant contributions to the protein structure and function predictive models.^16–22^ Alpha-fold is a fantastic example of AI success in protein structure prediction.^23–25^ However, there has been less focus on the structure-agnostic AI bioinformatic models that predict the higher-order interactions between amino acids using sequences. With the rise of large language models (LLMs) and transformers, there has been a renaissance in bioinformatics since protein sequences can be considered as language.^26–28^ LLMs can take advantage of transformer architecture and learn higher-order relations in texts.^21,29,30^ In this study, we leveraged the recent advances of LLMs in protein modeling and developed a model for interpreting G protein-coupled receptor (GPCRs) sequences. We developed GPCR-BERT which investigates the high-order interactions in GPCRs. The reason we chose GPCRs is that they are an essential class of cell membrane proteins found in organisms ranging from bacteria to humans.^31–39^ Currently, over one-third of U.S. Food and Drug Administration-approved medications target the receptors.^35,40–45^ GPCRs exhibit remarkable functional diversity, including sensory perception, neurotransmission, immune response, cardiovascular regulation, vision, metabolism, and energy homeostasis.^35,40,46–51^ Although each of the receptors has its own amino acid sequence, there are a few conserved motifs among them, such as NPxxY, CWxP, and E/DRY. Each conserved motif serves as a crucial component in the GPCRs^52–54^ and the type of each x can vary according to the receptor class. We posed critical questions regarding the sequence design of GPCRs including: (1) What is the correlation between variations in the conserved region of GPCRs (as xx in NPxxY, x in CWxP, and E/D in E/DRY) and other amino acids in the sequence? (2) Can we predict the whole sequence of amino acids in a GPCR, given a partial sequence? (3) Can we find amino acids having the most contributions to others that may play key roles in GPCR conformational changes? To answer these questions, we focused on three conserved motifs (NPxxY, CWxP, and E/DRY) in different types of receptors. We defined tasks to predict the variations in these motifs given the rest of the sequence and their correlations with other amino acids within the GPCR sequences using protein language models. The emergence of transformers has further enabled machine learning (ML) models to effectively capture long-range dependencies between words and gain a deeper comprehension of language syntax.^21,22^ In addition, the attention mechanism enables the model to weigh the importance of each element based on its contextual relationships within the sequence.^22^ Prot-Bert, a pretrained variant of bidirectional encoder representations from transformers (BERT), was employed for this research and fine-tuned with tokenized amino acid sequences. This approach facilitated the model’s learning of the intrinsic patterns present within the GPCR sequence.^21,55^ Furthermore, we interpreted attention weights and hidden states of the fine-tuned model to extract the extent of contributions of other amino acids in determining the type of masked amino acids (x and E/D) in the conserved motifs. Investigating the extent of contributions of amino acids in dictating the type of masked amino acids may assist us in identifying the amino acids correlated to the function of the conserved sequences. The correlated amino acids can serve as potential candidates for mutation studies and aid in the generation of new protein structures in protein engineering.

## Methods

**Figure 1:**
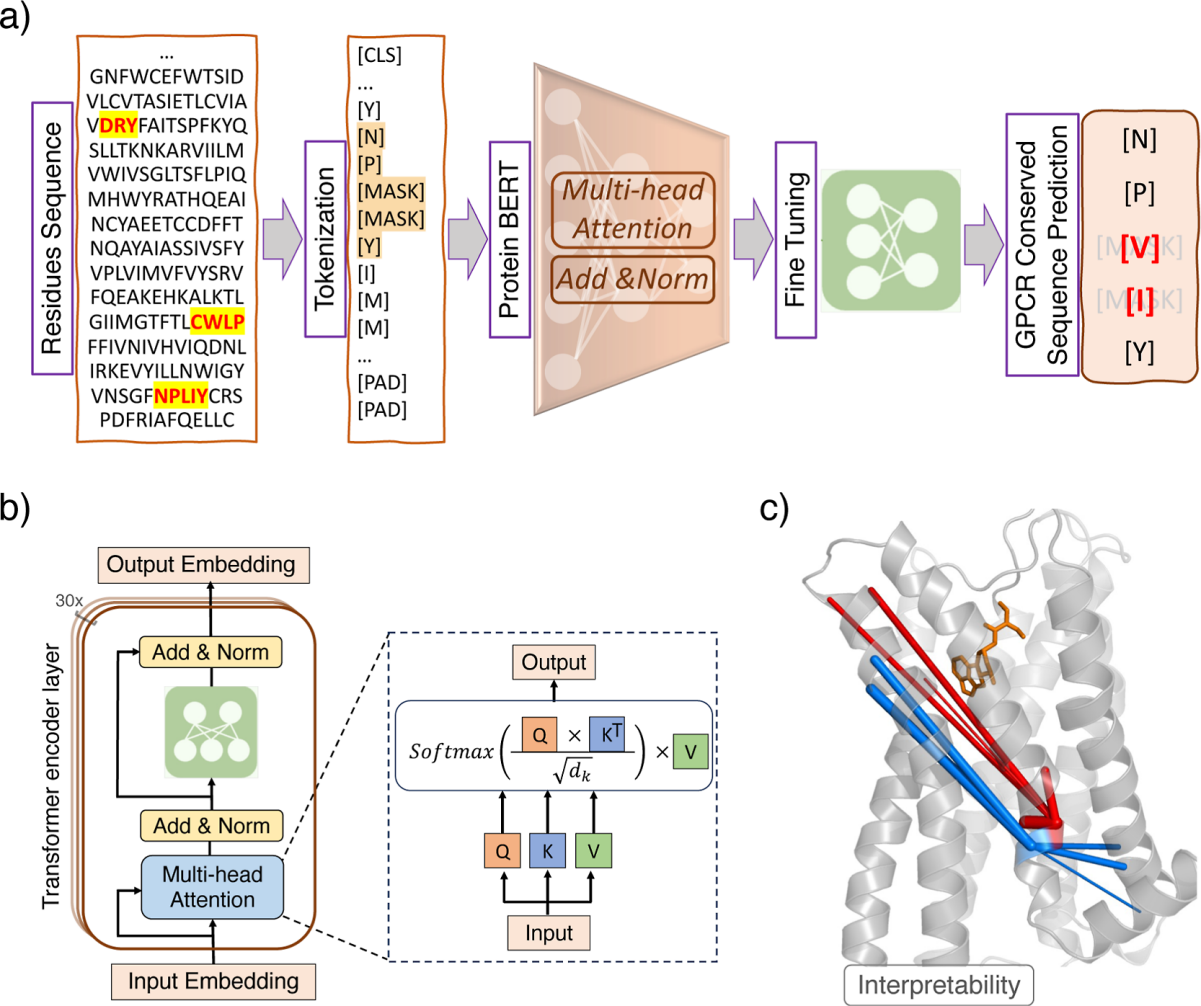
(a) Overall architecture of GPCR-BERT. Amino acid sequences are tokenized and subsequently processed through Prot-Bert, followed by the regression head. (b) Structure of Prot-Bert transformer and the attention layer. The input token embedding is transformed into keys, queries, and values which subsequently form the attention matrix. The output is passed through a fully connected neural network. This sequence of operations is iterated 30 times to reach the final output embedding of the GPCR sequence. (c) Representation of the top five most correlated amino acids to the first x (red) and second x (blue) in NPxxY motif within a GPCR obtained through attention heads. The thickness of the lines represents the strength of correlations (weights).

The overall architecture of GPCR-BERT is displayed in Figure 1a. Prot-Bert,^55^ a transformer-based model of 16-head and 30-layer structure, has been used as the pretrained model. Prot-Bert is pretrained on a massive protein sequence corpus, UniRef100,^56^ which contains over 217 million unique sequences. A variant of the original BERT^21^ developed by Google, Prot-Bert adopts the structure of the encoder segment of the transformer model, comprised of multiple, sequential layers of an attention-feed-forward network (Figure 1b). Within the attention structure, each token is encoded into input embedding, which is converted into keys, queries, and values. Keys and queries are combined via matrix multiplication to form the attention map, which is subsequently passed through a softmax^57^ function to generate a probability distribution. Following this process, the resulting distribution is used to scale (multiply) the value vectors. The feed-forward layer within each Transformer layer facilitates the learning of intricate patterns embedded in the input, whereas the attention mechanism is responsible for understanding and encoding the relationships among various tokens. The multihead attention structure divides the input across multiple parallel attention layers, or “heads”. This setup enables each head to independently learn and specialize in capturing different types of patterns and relationships.^22^ This structure of the Transformer encoder in Prot-Bert enables the model to learn context-aware representations of amino acids in a protein sequence by considering each sequence as a “document”. The model has been trained with a masked language model objective, which is a training method where some percentage of input tokens is masked at random, and the model must predict those masked tokens from their context. One of the biggest advantages of Prot-Bert over some other pretrained models (such as Prot-XL and Prot-Albert) is that its performance has exceeded these models on several benchmarks.^55^ For the fine-tuning process, a series of experiments were performed to identify the most suitable architecture for the regression head. As a result, the design of three fully connected layers is selected.

### Data Preprocessing

The GPCR sequences data set used for fine-tuning Prot-Bert was obtained from the GPCRdb database.^58^ The GPCRdb database serves as a comprehensive resource of valuable experimental data on GPCRs. This includes details on GPCR binding, configurations, and signal transduction. Moreover, the database offers a range of analysis tools and computational data, such as homology models and multiple sequence alignments, which further enhance the understanding and exploration of GPCRs.^58–60^ From the GPCRdb, all class A GPCR sequences that contain either NPxxY, CWxP, or E/DRY motifs are extracted, obtaining a data set of 293 protein sequences. Among the extracted protein sequences, some of them were incomplete sequences embodying missing residues that are denoted as “X” which were especially prevalent in relatively long sequences. Considering the distribution of sequence lengths (Figure S1), we knocked out the sequences that are larger than 370 which is 13.3% of the initial data. The aim was to remove those that contain excessive missing residues and avoid inefficient padding in the tokenization process. This filtering process led to a data set of 254 class A GPCR sequences distributed across 62 different receptor classes (Table S1). Although the three motifs are “highly” conserved, not every single class A GPCR contains these motifs. For example, residue C in the CWxP motif is only conserved 71% among class A GPCRs.^61^ Thus, the initial data set from GPCRdb was refined to 3 separate data sets to include only those sequences that embody each conserved motif. This process yielded a data set composed of 238 sequences for the NPxxY prediction task, 168 for the CWxP task, and 212 for the E/DRY task.

In the tokenization process, we searched through each sequence for the location of the conserved motifs, ensuring their proper localization within specific regions: NPxxY in Transmembrane TM7, CWxP in TM6, and E/DRY in TM3. The “x” residue earmarked for prediction (e.g., xx in the NPxxY) was substituted with “J”, an alphabet character absent from the Prot-Bert tokenizer vocabulary. Simultaneously, label sequences were assembled, each retaining the ground truth amino acid while substituting all other residues with “J”. During tokenization, the “J” characters in the input sequences were replaced with the [MASK] token. Utilizing the Prot-Bert tokenizer, a [CLS] token was inserted at the beginning of each sequence, while sequences falling short of the maximum input length were padded using the [PAD] token. Both the input and label sequences were then translated into the corresponding integers as dictated by the tokenizer vocabulary.

## Results

### Prediction Tasks of Conserved Motifs

**Figure 2:**
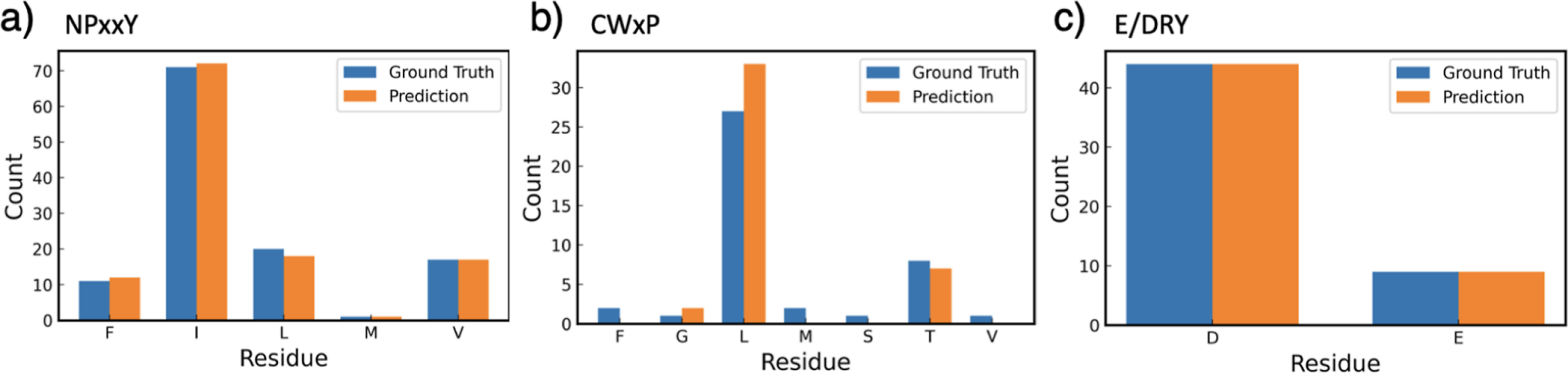
Test result for the downstream tasks of predicting variations in (a) NPxxY, (b) CWxP, and (c) E/DRY. Each task was subjected to training for 30 epochs prior to testing. For the NPxxY task, the prediction results for both masked positions were taken into account.

The curated data sets were partitioned into training and testing subsets to a ratio of 0.75:0.25 and were subsequently incorporated into the structure of Prot-Bert, complemented by a regression head. A series of experiments were performed to identify a suitable architecture for the regression head. The optimal architecture was chosen as the three fully connected layers of multilayer perceptrons with respective node counts of 1024, 256, and 30. This specific configuration yielded the highest prediction accuracy. Additionally, the model incorporated a dropout rate of 0.25 and the rectified linear unit^62^ activation function. An Adam optimizer^63^ was employed alongside a learning rate (LR) scheduler to facilitate the model training. The LR scheduler was designed to diminish the LR by a factor of 0.2, contingent on stagnation or a lack of improvement in the loss function. The loss function chosen for this task was a cross-entropy loss, which represents a composite of a softmax activation function and a negative log-likelihood loss function. This choice was particularly appropriate for the multiclass prediction task at hand, as it imposes more substantial penalties on the model when erroneous predictions are made with high confidence. Furthermore, multiclass accuracy was assessed by contrasting the output of the regression head with the label tokens. The model was trained on a single NVIDIA GeForce GTX 2080 Ti GPU with 12 GB of memory. The results of the fine-tuning process are delineated in Table 1 and Figure 2. The NPxxY task and E/DRY task demonstrated exceptional performance, achieving nearly perfect accuracy with minimal error. In comparison, the performance on the CWxP task was lower yet within an acceptable range of accuracy. This relative disparity in performance, particularly for the CWxP task, is assumed to be attributed to the smaller data set size compared to the other two tasks.

**Table 1:** Fine-Tuning Result of Each Prediction Task^a^

conserved motif	number of data	train loss	test loss	train accuracy	test accuracy
NPxxY	238	0.138 ± 0.020	0.213 ± 0.015	99.25 ± 0.323	98.05 ± 0.479
CWxP	168	0.376 ± 0.040	0.702 ± 0.040	89.94 ± 0.468	86.29 ± 1.010
E/DRY	212	0.092 ± 0.0006	0.089 ± 0.0006	100	100

a The outcomes pertaining to loss and accuracy for each downstream task are displayed. Each task is averaged over the results of three runs.

### Prediction Tasks of Masked Sequences

To evaluate GPCR-BERT’s capability to predict parts of the sequence other than the conserved motifs and to find out whether it is possible to infer a complete GPCR sequence from partial information, we have tested the model with several masked sequence prediction tasks, as shown in Table 2. The data set containing all 254 GPCR sequences is used since these tasks are not related to separate conserved motifs. For each GPCR sequence, residues for each task are continuously masked with “J” tokens starting from the sequence index 100 which is a randomly selected number. The number of masking is increased to 10, 15, and 50 to further examine the model’s prediction capability. The 5 masked prediction task shows the highest prediction accuracy of 83.07% while the task of 10, 15, and 50 shows similar accuracy of around 75%. The decent prediction accuracy indicates that GPCR-BERT is capable of predicting a large number of residues and not just a small number of residues in the conserved motifs.

**Table 2:** Result of Masked Sequence Prediction Task^a^

task	train loss	test loss	train accuracy	test accuracy
5 masked	0.174 ± 0.015	0.837 ± 0.040	99.26 ± 0.184	83.07 ± 0.661
10 masked	0.159 ± 0.026	1.193 ± 0.025	99.32 ± 0.127	74.57 ± 1.502
15 masked	0.154 ± 0.021	1.166 ± 0.015	99.31 ± 0.336	76.72 ± 0.440
50 masked	0.134 ± 0.010	1.254 ± 0.056	99.58 ± 0.233	74.89 ± 1.115

a The outcomes pertaining to loss and accuracy for each downstream task are displayed. Each task is averaged over the results of three runs.

### Comparison with Other Models

To compare the GPCR-BERT’s performance with other ML models, we tested several prediction tasks to the original BERT and the support vector machine (SVM). Compared with BERT, we can investigate the effect of leveraging an encoder pretrained on protein sequences as opposed to one trained on a generic English corpus. By testing on SVM, we can investigate how low-key ML models can perform when trained with limited protein sequence data. SVM is a popular supervised learning algorithm that is known for its high accuracy, ability to handle high-dimensional data, and versatility in modeling both linear and nonlinear relationships. SVM works by finding a hyperplane that best divides the data set into classes. The main objective of the SVM is to maximize the margin between data points and the separating hyperplane. The best hyperplane is the one for which this margin is maximized.^64^

The data set used for all models has the same train/test split ratio and random state. As shown in the result (Table 3), BERT was able to predict quite well (83% accuracy) in a relatively simple E/DRY task, but the accuracy decreased significantly for more intricate tasks like NPxxY and the 5 masked prediction. From this, we can confirm the advantage of utilizing pretrained models on protein database corpus in the interpretation of protein sequences. Interestingly, although the performance was incomparable to the GPCR-BERT, SVM has shown better results than the original BERT. This suggests that BERT’s pretraining on English text may have adversely affected the learning of protein sequences.

**Table 3:** Test Accuracy (%) of Each Prediction Task of Different ML Models^a^

model	E/DRY	NPxxY	CWxP	5 masked
GPCR-BERT	100	98.05 ± 0.479	86.29 ± 1.010	83.07 ± 0.661
BERT	83.02	59.85 ± 0.266	71.43	10.97 ± 0.312
SVM	90.57	76.67	71.43	63.49

a Each task is averaged over the results of three runs.

### Class Exclusion Validation

**Figure 3:**
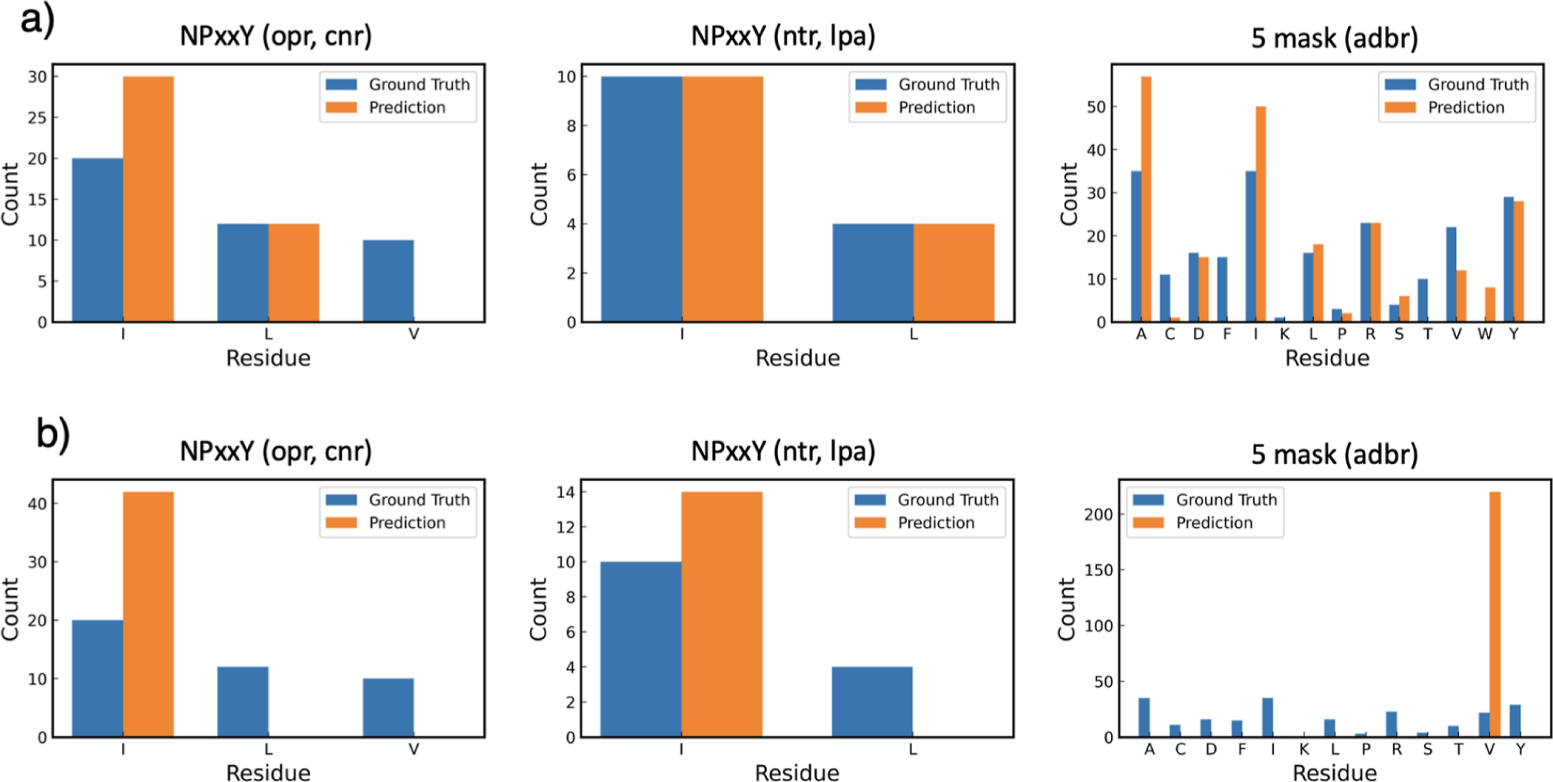
Test result for the class exclusion validation tasks, starting from the left, NPxxY (excluding opr, cnr), NPxxY (excluding ntr, lpa) and 5 masks (excluding adbr). Tasks were compared for GPCR-BERT (a) and original BERT (b). Each task was subjected to training for 30 epochs prior to testing.

The random split of the training and test set might lead the GPCR-BERT to take advantage of the sequence information on the identical receptor class. For example, if “5DHH” GPCR, an opioid (opr) class receptor, was in the training set, the model might exploit this information to predict the conserved motifs of the “5DHG” in the test set, which is also an opr class receptor with similar structure. To assess GPCR-BERT’s predictive capability independent of information from identical classes, we excluded certain classes from the training set, allocating them exclusively to the test sets. For the first NPxxY task, opr and cannabinoid (cnr) receptor classes were excluded, while for the second task, neurotensin (ntr) and lysophosphatidic acid (lpa) classes were not included. The model’s performance, as shown in Table 4, and the prediction results, depicted in Figure 3, indicate that while the original BERT model tended to predict only the most common ground truth residue (I for NPxxY tasks and V for the 5 mask task) without differentiating between protein structures across receptor classes, GPCR-BERT demonstrated a markedly improved ability to identify correct conserved motifs despite the exclusion of these classes in the training phase. With the 5 mask prediction task, GPCR-BERT also exceeded BERT in order of magnitude in exclusion of the adreno β (adbr) receptor class, illustrating its enhanced proficiency in understanding GPCR sequences.

**Table 4:** Results of Class Exclusion Validation Task^a^

task (exclusion)	train loss	test loss	train accuracy	test accuracy	train accuracy (BERT)	test accuracy (BERT)
NPxxY (opr, cnr)	0.200 ± 0.017	1.172 ± 0.198	97.62 ± 0.584	76.19	58.45 ± 0.139	47.62
NPxxY (ntr, lpa)	0.160 ± 0.005	0.106 ± 0.005	98.41 ± 0.497	100.00	57.00 ± 0.121	71.43
5 mask (adbr)	0.438 ± 0.021	1.880 ± 0.039	94.46 ± 1.155	48.03 ± 2.147	11.12 ± 1.073	11.97 ± 3.412

a The outcomes pertaining to loss and accuracy for each downstream task are displayed. Each task is averaged over the results of three runs.

## Discussion

### Interpretability

**Figure 4:**
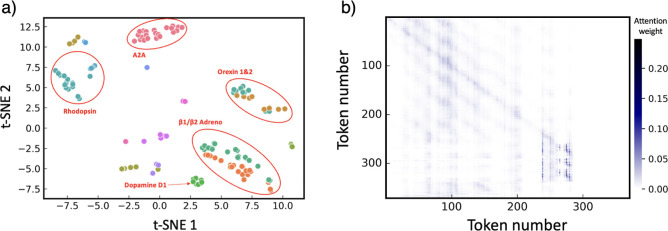
(a) t-SNE visualization of GPCRs. t-SNE was applied to the [CLS] token embedding from the last hidden state of GPCR-BERT to reduce the dimension to 2D. Different receptor classes are denoted by distinct colors and the clustering of the same GPCR type is obvious. (b) Attention weight heatmap of human β 2 adreno receptor GPCR (4GBR). The figure provides a visual representation of head 1 of the final attention layer in GPCR-BERT. The labels on the axes correspond to the respective tokens.

**Figure 5:**
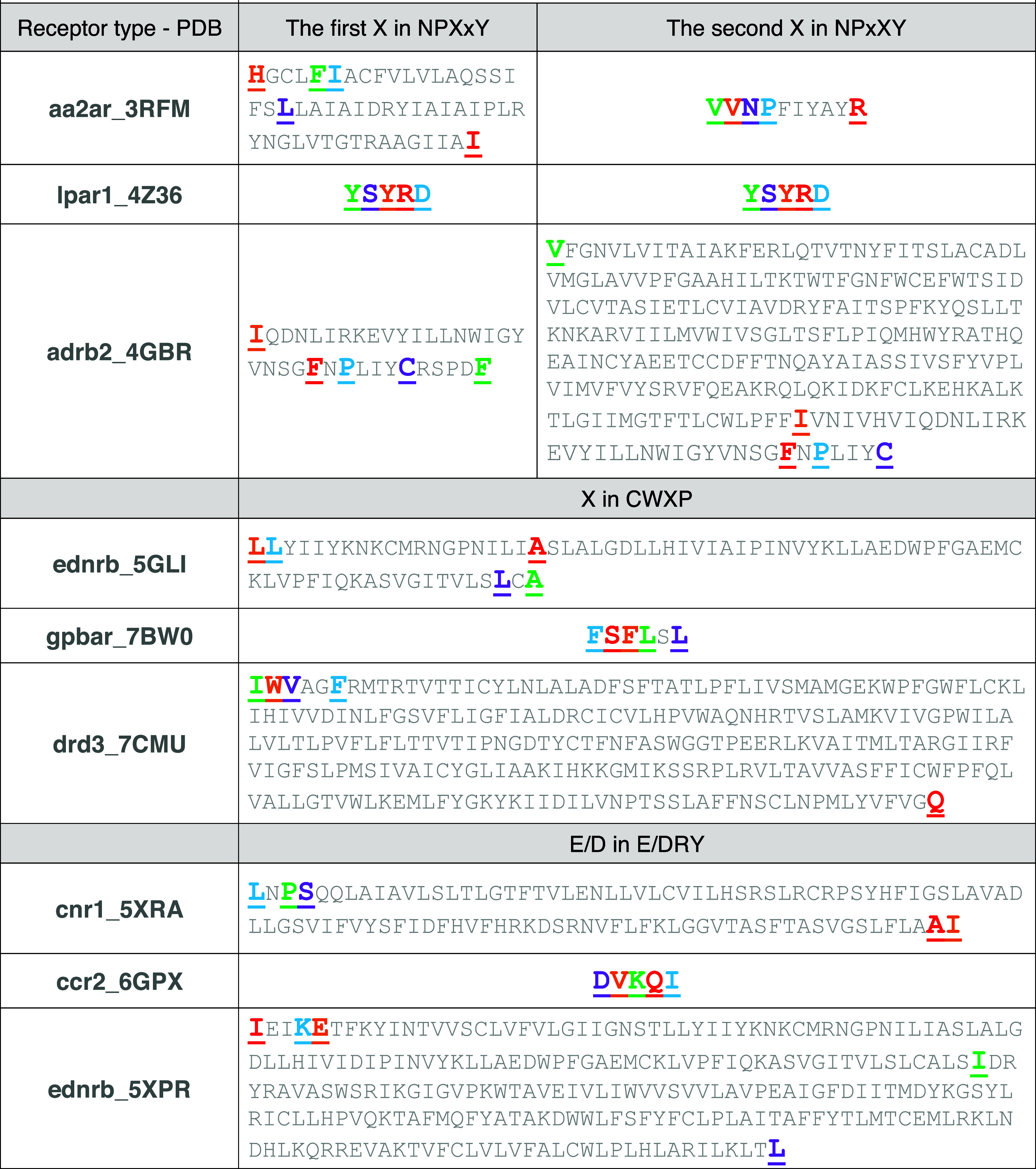
Top five [first (red), second (orange), third (blue), fourth (green), and fifth (purple)] most correlated amino acids to the masked amino acids in NPxxY, CWxP, and E/DRY motifs in various GPCRs.

**Figure 6:**
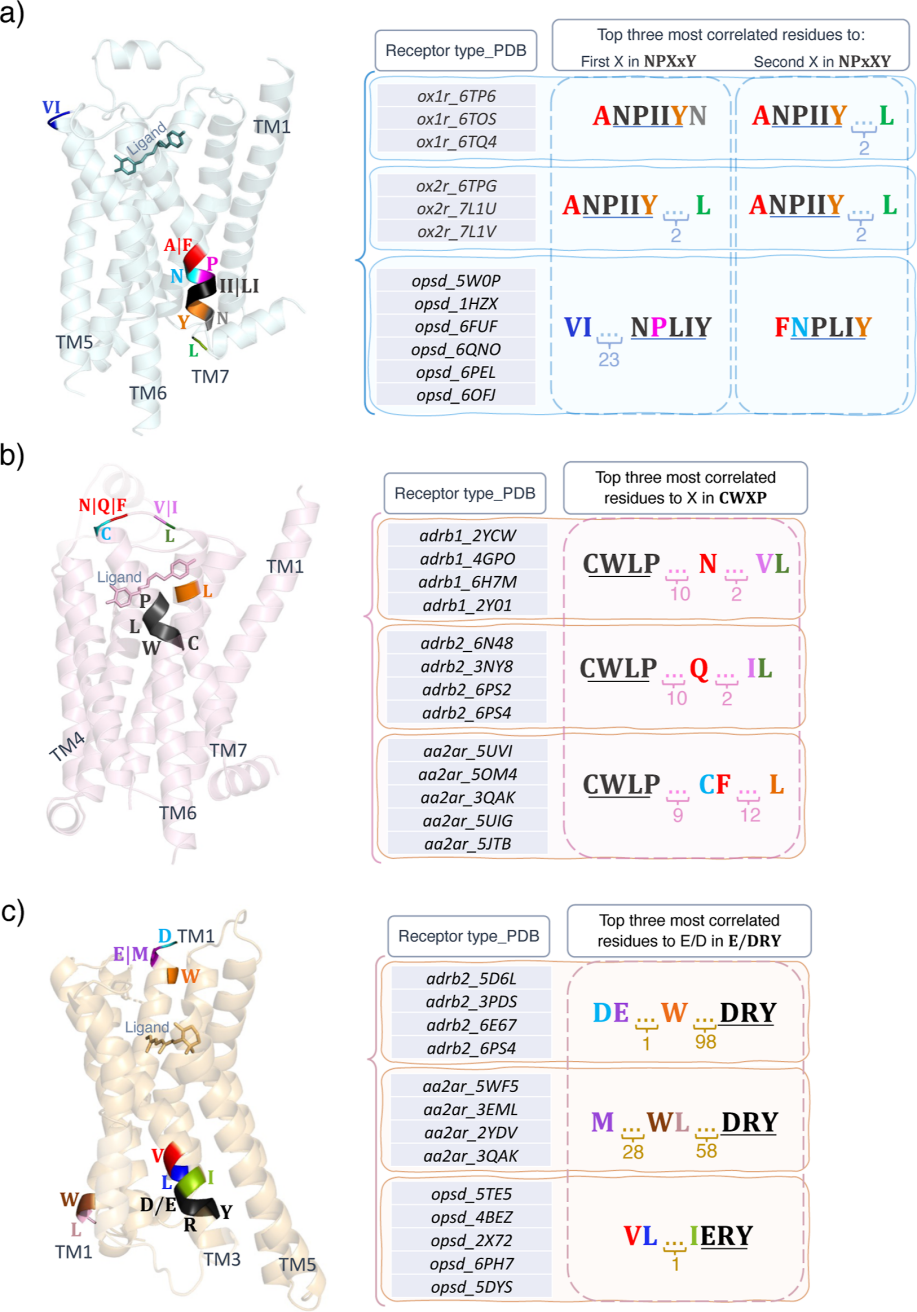
Structural representations for the top three most correlated amino acids to (a) xx in NPxxY, (b) x in CWxP, and (c) E/D in E/DRY motifs within various GPCRs. In the tables, the colors of the correlated amino acids correspond to the colors assigned to amino acids across the protein and the numbers denote the number of residues separating them.

The attention mechanism of the transformer model facilitates the capture of global sequence information within each token embedded in the encoder. Yet, in practical applications, the classification token, known as the [CLS] token, often serves as the aggregate representation of the entire sequence.^65,66^ The BERT tokenizer introduces the [CLS] token at the commencement of every sequence, which is then employed by BERT to assign the sequence to specific classes. Thus, the [CLS] token encapsulates information from all output embedding. Using this insight, we aimed to verify whether GPCR-BERT effectively recognizes and differentiates among the various GPCR types. To this end, we extracted the [CLS] tokens of each GPCR sequence from the final hidden state which has the structure of 1024 nodes and visualized with t-distributed stochastic neighbor embedding (t-SNE).^67^ t-SNE executes dimensionality reduction by determining pairwise similarities in high-dimensional space and constructing a Gaussian joint probability distribution. Correspondingly, a similar probability distribution is defined using a Student’s *t*-distribution. The algorithm then seeks to minimize the Kullback–Leibler divergence between these two distributions. This minimization process inherently attracts similar data points toward each other while repelling dissimilar points, thereby enabling the clustering of similar data points. The results are shown in Figure 4a. Interpreting the attention weights allows us to examine how the GPCR-BERT can understand the grammar of GPCR sequences. Within the hierarchical structure of multiple attention layers in the encoder, the terminal layer is most likely to capture advanced semantic information in the context of natural language processing tasks, and analogous structural or binding information for protein sequences.^21,68^ Thus, the 16 attention heads of the last layer of GPCR-BERT are extracted and visualized through heatmaps (Figure 4b). High attention weights are indicative of a greater degree of correlation between the corresponding residues in the input sequence. These elevated attention weights highlight specific regions of the sequence that the model perceives as containing valuable information. Attention allows every token in the input sequence to have a direct connection with all other tokens in the sequence, thereby facilitating the modeling of interdependencies between tokens irrespective of their relative positions.^22^ In addition, the softmax function of the attention enables the attention weight to serve as a probability distribution, resulting in the sum of each row being 1. Thus, the attention weights of GPCR-BERT represent the relative correlation of the corresponding residue with others. To scrutinize the interrelationships between conserved motifs and the remaining sequence in GPCRs, the five highest weights associated with variant residues within the conserved motifs are examined. In the context of the CWxP prediction downstream task, for instance, the attention weight corresponding to “x” is selected, and the top five values are analyzed for every GPCR sequence. The five residues displaying the highest correlation are further investigated using PyMOL^69^ to examine 3D crystal structure and the biological relevance of GPCR-BERT’s interpretation of GPCRs. The findings of this analysis are presented in Figures 5 and 6.

### Mapping Higher Order Interactions

The fine-tuned GPCR-BERT representations of GPCRs are depicted via a t-SNE plot (Figure 4a). For the sake of effective visualization, only classes consisting of more than three GPCRs were selected. As evidenced by the t-SNE plot, GPCRs of the same receptor class (depicted by identical color markers) are clustered together, thereby indicating the model’s successful classification of GPCRs based solely on their sequence information. This result also suggests that the [CLS] token within the embedding effectively captures the distinguishing characteristics of the individual GPCR classes. The model’s ability to differentiate between classes such as the Orexin1 and Orexin2 receptor and the β1/β2 Adreno receptor, both of which possess the NPIIY motif, implies that the model takes into account the entire GPCR sequence, rather than merely the conserved motifs when executing prediction tasks. Moreover, given that the intercluster distances within the t-SNE plot signify the degree of similarity between each cluster, it is evident that the GPCR-BERT is able to discern the subtle variations of GPCR classes. This capability is particularly notable in the case of distinguishing between β1 and β2 Adreno receptor classes depicted in the lower right corner of Figure 4a. A more detailed version of this plot can be found in Figure S2. Figure 4b illustrates the attention maps of the tokenized GPCR (4GBR) sequence as processed by the last layer of GPCR-BERT. The observed pattern of attention weight distribution remained consistent across the GPCR receptor class (refer to Figures S3 and S4 for heatmap of all 16 heads), suggesting that GPCR-BERT was successful in learning representations that underscore the interrelations of tokens within the sequence. As shown in Figure 4b, the padding region (far right) received an attention weight of 0, while a noticeably strong attention weight was detected and visualized in dark colors. This indicates that GPCR-BERT identified these residues as being more significantly correlated than others. This pattern was consistently observed across several heads (2, 3, 6, 8, 12, and 16) of 4GBR, and extended to other receptor classes of GPCRs. Notably, this pattern was particularly prevalent in heads 1 and 2, where the attention weights exceeded 0.1 in the corresponding region. Therefore, an analysis of these patterns is expected to yield valuable insights into the conserved motifs.

Figure 5 introduces the top five most correlated amino acids to the x residues in the NPxxY and CWxP motifs as well as the E/D residue in the E/DRY motif for a few receptors in the data set (see data availability for the comprehensive list of GPCRs and their correlated residues). The common pattern found for attention and consequent correlation between the NPxxY motif and other residues is that the xx residues mainly attend to their adjacent amino acids (NP). The first x in NPxxY usually attends to the upper top region of the receptors in the extracellular segment of protein. This higher-order interaction possibly demonstrates the connection between the NPxxY motif and the ligand-selective region of the receptor.^70^ This is significant because the xx residues vary between different types of GPCRs. Our finding elucidates why changes in the GPCR type correlate to xx residues in NPxxY. Figure 6 shows structural representations for the top three most correlated amino acids to the x in NPxxY (a) and CWxP (b) motifs and E/D in the E/DRY motif within various receptors. The correlated amino acids are displayed in colors and the numbers indicate the number of amino acids separating them. As the figure shows, similar types of receptors are categorized based on the correlated amino acid predictions. As noted earlier, the xx in NPxxY motif mostly attends to neighboring residues and some residues at the extracellular part of the receptor that are responsible for ligand selectivity. However, the x in the CWxP motif and E/D in the E/DRY motif mainly attend to some residues in the binding pocket. We can conclude that the message passed through the ligand in the binding pocket may be modulated by the variations in x and E/D residues within the NPxxY, CWxP, and E/DRY motifs. This finding will help us to focus on the sequential design of these motifs in selecting the ligand.

Furthermore, we investigated whether residues with high correlation, other than those adjacent to the conserved motifs, align with the experimental mutation findings. Thus, our correlation findings were compared with the mutagenesis data in the GPCRdb.^58^ These data contain the accumulated results of the positions of mutated residues and their effects on GPCRs. Table 5 shows the results for the comparison of the β 2 adreno receptor (results for other classes are shown in Tables S2–S4). It can be observed that heads 6 and 10 of the GPCR-BERT found the residues that are related to receptor expression significant while heads 11 and 13 discerned the significance of residues linked to thermostabilization. We can also see that the multihead attention mechanism of transformer models enables each head to learn distinct patterns and this characteristic can facilitate protein sequence analysis. Additionally, some highly correlated residues, such as residue E 3.26 in head 10, were identified by GPCR-BERT as potentially significant but remain understudied. These insights may provide a foundation for further investigations into GPCR structures and their mechanisms. There are, however, limitations to this study considering the minor inconsistency of the conserved motifs in class A GPCRs. As mentioned earlier, residue C in the CWxP motif is only conserved 71%^61^ while the NPxxY motif is conserved 94% among class A GPCRs. The remaining variations are in NPxxxY (3%) or NPxxF (3%) form.^71^ Similarly, Y in the E/DRY motif has exceptions as well.^72^ Since the model is focused on predicting only the general type of these motifs, its understanding of GPCRs might not apply to ones that have varied versions of the motifs.

**Table 5:** Comparison of GPCR-BERT Correlation Results with the Mutagenesis Data in GPCRdb for β 2 Adreno Receptors^a^

head	repetition	residue	location (BW)	matching with mutagenesis data
2	16	D in DFRIAF	8.49 (H8)	no match
4	18	L in LPFFIV	6.49	no match
4	15	G in GIIMGT	6.38	no match
4	17	H in HVIQDN	6.58	no match
5	9	K in KFERLQ	1.59	no match
7	11	T in TKTWTF	2.66	T 2.66 (receptor-expression)
7	17	I in IQMHWY	4.61	no match
11	11	K in KTWTFG	2.67	1 before T 23.49 (receptor-expression)
11	19	E in EFWTSI	3.26	no match
11	12	K in KEHKAL	6.28	2 after C 6.27 (thermostabilization)
11	9	E in EAKRQL	5.64	no match
11	11	E in ERLQTV	12.48 (ICL 1)	no match
12	6	I in ILTKTW	2.64	1 after H 2.63 (receptor–ligand-bond)
12	14	G in GAAHIL	2.6	3 before H 2.63 (receptor–ligand-bond)
14	9	C in CLKEHK	6.27	C 6.27
14	7	K in KFCLKE	6.25	2 before C 6.27 (receptor–ligand-bond)
14	6	L in LAVVPF	2.55	no match
15	20	R in RQLQKI	5.67	no match
15	19	R in RVFQEA	5.6	no match
15	6	K in KIDKSE	5.71	no match
16	17	H in HVIQDN	6.58	no match
16	7	P in PFGAAH	2.58	no match

a “Head” and “repetition” columns display the respective head number that detected the correlation and the frequency of its occurrence. “Residue” and “location” columns represent the residue and its position as per the Ballesteros–Weinstein (BW) numbering system. The “matching with mutagenesis data” column indicates if the identified correlation aligns with experimental mutation results.

## Conclusions

In this study, we explored the higher-order interactions in the sequence design of GPCRs and their impact on determining the type of function by taking advantage of language models. We utilized the Prot-Bert model, a transformer-based language model, and fine-tuned it with tokenized amino acid sequences to predict nonconserved amino acids (x, E/D) in the conserved motifs of NPxxY, CWxP, and E/DRY in various GPCRs. The results showed that the model effectively predicted these residues in the receptors with a high accuracy. The attention weights and hidden states of the model were also analyzed to understand the extent of contributions of other amino acids in dictating the type of masked ones. We demonstrated that GPCR-BERT successfully distinguished different GPCR classes based on their sequences and has the potential for understanding functional relationships within protein structures. These findings could also have implications in mutation studies, protein engineering, drug development, and further advances in our understanding of GPCR biology.

## Data Availability

The necessary information containing the codes and data for downstream tasks used in this study is available here: https://github.com/Andrewkimmm/GPCR-BERT.

## Supporting Information Available

The supporting information includes a complete figure of the t-SNE of GPCR-BERT and heatmaps visualizing all 16 attention heads of several GPCRs (PDF)
