# Supplementary material for: GPCR-BERT: Interpreting Sequential Design of G Protein-Coupled Receptors Using Protein Language Models
Source: J Chem Inf Model. 2024 Feb 10;64(4):1134–44. doi: 10.1021/acs.jcim.3c01706 (PMC10900288; doi:10.1021/acs.jcim.3c01706)
Supplement: Supplementary file 1 — ci3c01706_si_001.pdf [file ci3c01706_si_001.pdf]

Supporting Information: GPCR-BERT: Interpreting Sequential Design of G Protein Coupled Receptors Using Protein Language Models

Seongwon Kim,[†,‖] Parisa Mollaei,[‡,‖] Akshay Antony,[‡] Rishikesh Magar,[‡] and Amir Barati Farimani*,[‡,¶,§]

†*Department of Chemical Engineering, Carnegie Mellon University, 15213, USA*

‡*Department of Mechanical Engineering, Carnegie Mellon University, 15213, USA*

¶*Department of Biomedical Engineering, Carnegie Mellon University, 15213, USA*

§*Machine Learning Department, Carnegie Mellon University, 15213, USA*

‖*Joint First Authorship*

E-mail: barati@cmu.edu

Data distribution of initial dataset

Fig.S1 displays the distribution of GPCR data sequence lengths that are initially extracted from GPCRdb and after filtering process. 293 Class A GPCRs that embody either NPxxY, CWxP or E/DRY motifs are extracted from the GPCRdb (Fig.S1(a)). To avoid sequences with excessive missing residues and ensure efficient tokenization, we excluded sequences exceeding 370 residues leading to a filtered dataset of 254 sequences (Fig.S1(b)). This dataset consists of 62 receptor classes and distribution is displayed in Table S1.

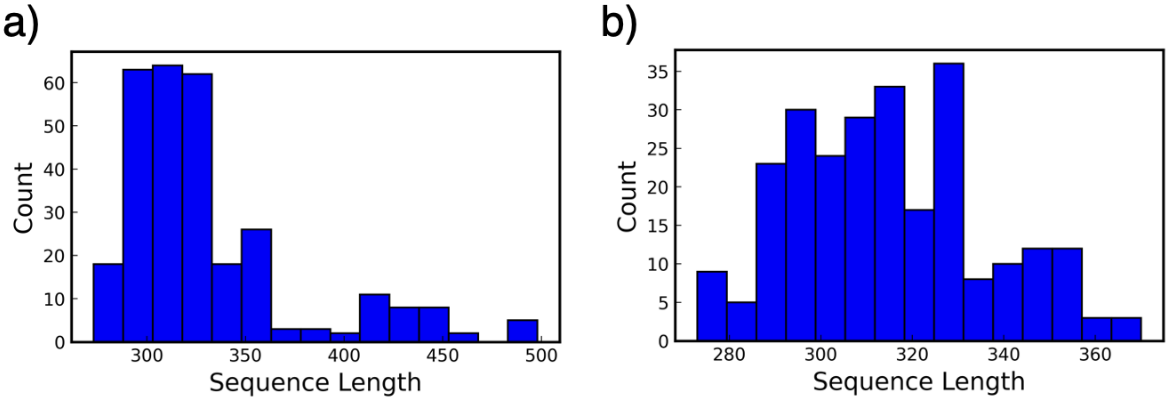

Figure S1: Distribution histogram of the sequence length of GPCRs in the initial dataset (a) and after filtering (b).

Table S1: Receptor class distribution of dataset, only classes that have more than 3 data are displayed. The dataset consists of 62 receptor classes with 44 of them having less than 4 data.

aa2ar	24	drd1	9	cnr1	5	5ht2a	4
adrb1	23	ox2r	8	cnr2	4	mtr1a	4
adrb2	21	5ht2b	8	ntr1	4	ebnrb	4
opsd	20	nk1r	6	cltr2	4	others	83
ox1r	14	cxcr4	5	gpr52	4	total	254

Fully Labeled t-SNE

Fig.S2 displays the fully labeled t-SNE plot from GPCR-Bert with distinct colors representing different receptor classes. [CLS] token embeddings are extracted from the last hidden state and visualized. Note that from the complete dataset, only classes comprising more than three data points have been taken into account. Notably, even classes with a small number of data points are clearly distinguished. For example, GPCR-Bert effectively recognizes the subtle sequential difference between CNR1 and CNR2 receptor classes (upper left corner).

Attention head heatmap

Heatmaps in Fig.S3 and Fig.S4 present a graphical illustration of the last attention layer in GPCR-Bert. Attention weights of all 16 heads are extracted and visualized, with darker

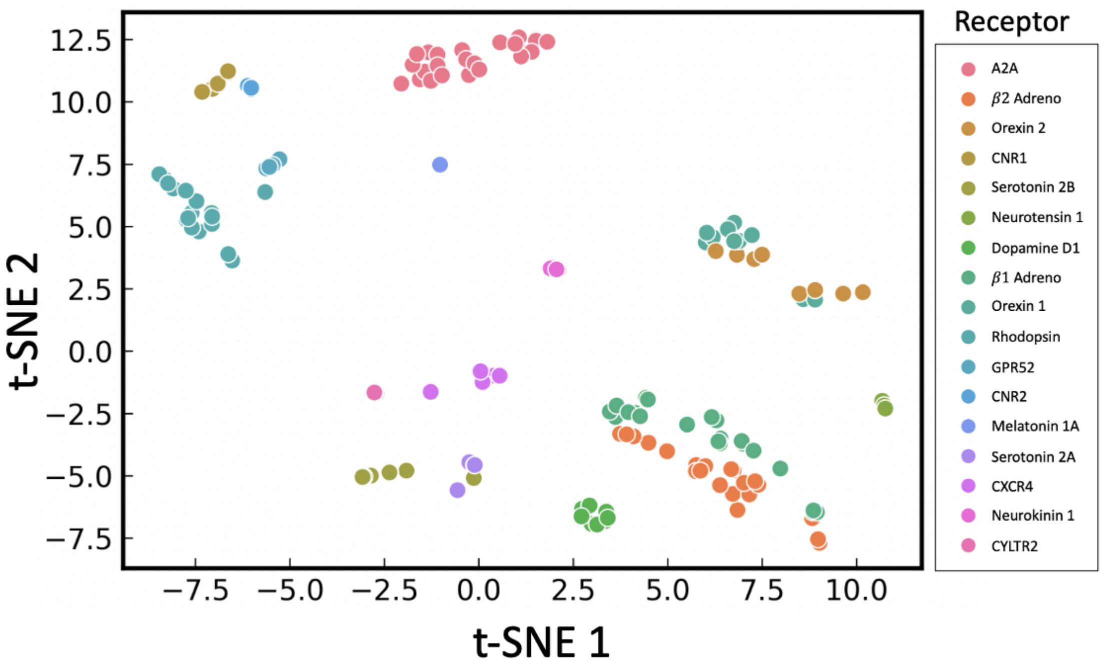

Figure S2: Fully labeled t-SNE of GPCRs. The [CLS] token embedding from the last hidden state has been subjected to both dimension reduction and clustering techniques.

colors representing larger weights. The multi-head attention mechanism allows the model to capture distinct patterns of different parts of the input embeddings as each head is assigned to investigate different parts. A comparative analysis of the heatmaps reveals that GPCR-Bert gave a similar interpretation of sequences according to their classes comparing Fig.S3 (β adreno receptor class) with Fig.S4 (Dopamine 1 receptor class). In addition, several heads such as Head 1 and Head 2 displays similar pattern irrespective of different GPCR classes.

Comparison with Mutagenesis data

Table S2 to S4 shows the comparison with the correlation results of the GPCR-BERT and the mutagenesis data from the GPCRdb. The top 5 residues with the highest attention weights were identified from the final layer of GPCR-BERT and compared with the experimental mutagenesis results in GPCRdb. From the results, we can observe that some portion of the residues that GPCR-BERT identified as significant residues align with the experimental

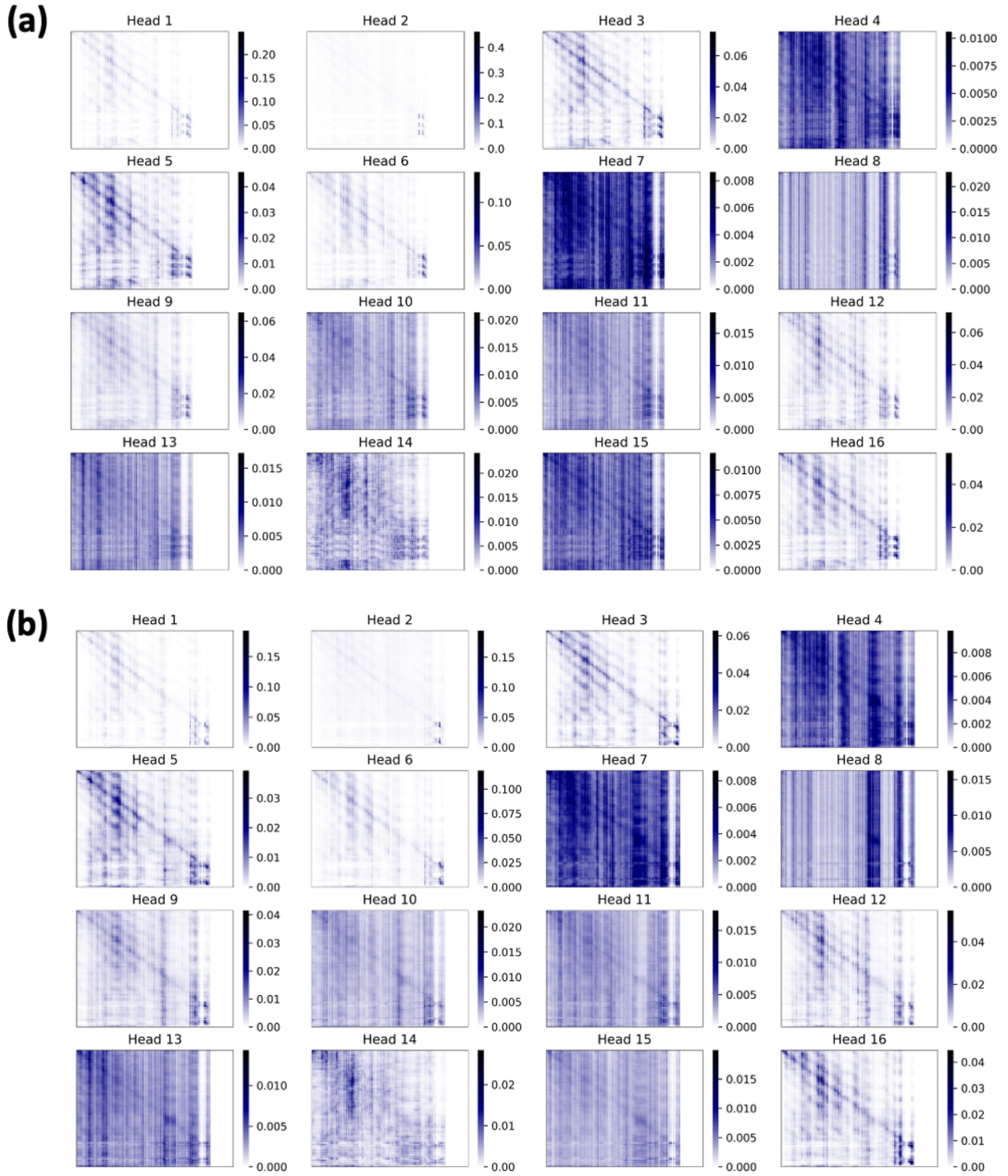

Figure S3: Attention weight heat map of (a) β 2 adreno receptor GPCR(4GBR), (b) β 1 adreno receptor GPCR(4AMI). The figure provides a visual representation of the final attention layer.

mutation studies.

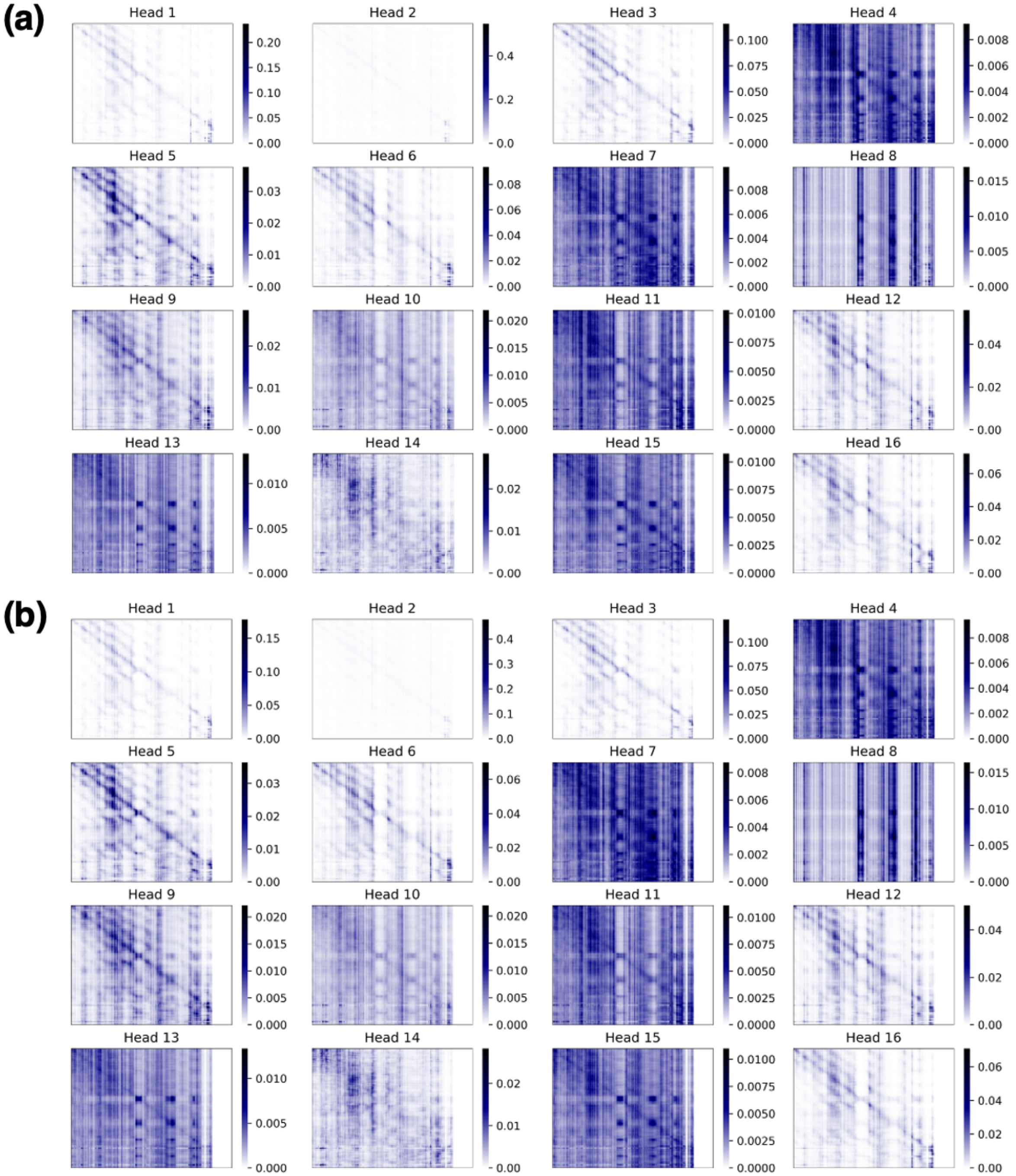

Figure S4: Attention weight heat map of (a) Dopamine 1 receptor GPCR(7LJC) and (b) Dopamine 1 receptor GPCR(7CKX). The figure provides a visual representation of the final attention layer.

Table S2: Comparison of GPCR-BERT correlation results with the mutagenesis data in GPCRdb for A2a receptors. 'Head' and 'Repetition' columns display the respective head number that detected the correlation and the frequency of its occurrence. 'Residue' and 'Location' columns represent the residue and its position as per the Ballesteros-Weinstein (BW) numbering system. The 'Matching with Mutagenesis data' column indicates if the identified correlation aligns with experimental mutation results.

**Head**	**Repetition**	**Residue**	**BW**	**Matching with Mutagenesis data**
4	4	K in KGIIAI	4.43	**K 4.43 (thermostabilization)**
4	4	K in KSAAII	6.35	**2 before A 6.37 (thermostabilization)**
4	24	H in HAAKSL	6.32	**5 before L 6.37 (thermostabilization)**
4	7	K in KGIIAI	4.43	**K 4.43 (thermostabilization)**
5	20	S in SLLAIA	3.42	**3 after S 3.39 (thermostabilization)**
6	24	F in FCPDCS	6.60	**no match**
7	19	T in TLQKEV	6.25	no match
8	9	A in APLWLM	7.30	**no match**
9	24	H in HGCLFI	3.23	no match
9	24	I in IACFVL	3.28	**no match**
9	12	F in FIACFV	3.27	no match
10	24	H in HAAKSL	6.32	**5 before L 6.37 (thermostabilization)**
10	20	K in KEVHAA	6.29	**no match**
11	8	K in KGIIA	4.43	**K 4.43 (thermostabilization)**
11	16	R in RAKGII	4.41	**2 before K 4.43 (thermostabilization)**
11	18	Q in QKEVHA	6.28	**no match**
12	24	F in FVVSLA	2.42	**4 before L 2.46 (thermostabilization)**
12	24	Y in YFVVSL	2.41	**5 before L 2.46 (thermostabilization)**
13	24	T in TGTRAK	4.38	**5 before K 4.43 (thermostabilization)**
13	22	T in TGTRAK	4.36	no match
13	18	A in ACLFED	4.69 (End of TM4)	no match
13	20	A in APLWL	7.30	no match
14	24	L in LRYNGL	34.51 (ICL2)	no match
15	8	H in HGCLFI	3.23	no match
15	17	L in LAARRQ	5.63	**L 5.63 (thermostabilization)**
16	23	I in IPFAIT	2.57	**5 before T 2.62 (thermostabilization)**
16	7	V in VSLAA	2.44	**2 before L 2.46 (thermostabilization)**

Table S3: Comparison of GPCR-BERT correlation results with the mutagenesis data in GPCRdb for β 1 Adreno receptors. 'Head' and 'Repetition' columns display the respective head number that detected the correlation and the frequency of its occurrence. 'Residue' and 'Location' columns represent the residue and its position as per the Ballesteros-Weinstein (BW) numbering system. The 'Matching with Mutagenesis data' column indicates if the identified correlation aligns with experimental mutation results.

**Head**	**Repetition**	**Residue**	**BW**	**Matching with Mutagenesis data**
1	8	R in RYQSL	34.52 (ICL 52)	no match
1	15	T in TLVVRG	2.62	no match
3	6	K in KEQIRK	5.56	no match
3	6	D in DFRKAF	8.49 (H8)	no match
3	8	Y in YCRSPD	7.53	**Y 7.53 (thermostabilization)**
4	18	C in CRSPDF	7.54	**1 after Y 7.53 (thermostabilization)**
6	15	F in FKRLLA	8.54 (H8)	**5 before A 8.59 (palmitoylation-site)**
7	6	I in IGSTQR	1.57	**2 before S 1.59 (thermostabilization)**
10	9	E in EQIRKI	5.67	no match
10	7	R in RKIDR	5.70	no match
11	9	Q in QIRKID	5.68	no match
11	9	E in EQIRKI	5.67	no match
11	17	Q in QRLQTL	12.48 (ICL 1)	**2 after R 1.59 (thermostabilization)**
12	15	R in RYQSLM	34.52 (ICL 2)	no match
12	11	K in KVIICT	4.43	no match
12	9	R in RARAKV	4.39	no match
12	14	Q in QIRKID	5.68	no match
13	13	V in VWAISA	4.49	no match
13	14	T in TSPFRY	3.55	no match
13	17	T in TNRAYA	5.34	no match
13	10	D in DFRKAF	8.49 (H8)	no match
14	9	K in KVIICT	4.43	no match
14	6	N in NLFITS	2.40	no match
15	12	R in RKIDR	5.70	no match
15	9	R in RAKVII	4.41	no match
15	7	R in RSPDFR	7.55	no match
16	9	P in PFGAT	2.58	**5 after M 2.53 (thermostabilization)**

Table S4: Comparison of GPCR-BERT correlation results with the mutagenesis data in GPCRdb for Rhodopsin receptors. 'Head' and 'Repetition' columns display the respective head number that detected the correlation and the frequency of its occurrence. 'Residue' and 'Location' columns represent the residue and its position as per the Ballesteros-Weinstein (BW) numbering system. The 'Matching with Mutagenesis data' column indicates if the identified correlation aligns with experimental mutation results.

**Head**	**Repetition**	**Residue**	**BW**	**Matching with Mutagenesis data**
1	11	E in ESFVIY	5.36	no match
1	13	L in LLIMLG	1.41	no match
2	18	Q in QFRNCM	8.49 (H8)	no match
2	13	C in CMVTTL	8.53 (H8)	no match
3	18	Q in QFRNCM	8.49 (H8)	**no match**
3	16	C in CMVTTL	8.53 (H8)	**no match**
4	13	G in GGFTTT	2.55	**1 before G 2.56 (INCL disease causing)**
4	13	G in GFTTTL	2.56	**G 2.56 (INCL disease causing)**
4	18	G in GGEIAL	3.35	no match
4	13	V in VVCKPM	3.53	no match
4	15	G in GQLVFT	5.59	no match
5	18	L in LYVTVQ	4.54	no match
5	17	G in GFTTTL	2.56	**G 2.56 (INCL disease causing)**
5	18	F in FTTTLY	2.57	**1 after G 2.56 (INCL disease causing)**
6	15	L in LICWLP	6.45	**5 after M 6.40 (receptor expression)**
6	15	C in CMVTTL	8.53 (H8)	no match
7	14	T in TTTLYT	2.56	**2 after G 2.56 (INCL disease causing)**
7	18	V in VVCKPM	3.53	no match
7	18	V in VVVCKP	3.52	no match
7	18	Y in YVVVCK	3.51	no match
7	18	T in TLYVTV	1.53	no match
8	15	A in APPLVG	4.58	no match
8	18	G in GFPINF	1.46	no match
8	18	A in AFTWVM	4.47	no match
8	11	A in AIERYV	2.47	no match
8	14	S in SNFRFG	34.52	no match
9	14	E in EGFFAT	3.28	**E 3.28 (receptor expression)**
9	18	L in LGGEIA	3.34	no match
9	12	E in EIALWS	3.37	no match
9	13	Q in QFRNCM	8.49 (H8)	no match
10	18	F in FCYGQL	5.56	no match
10	18	R in RYVVVC	3.50	no match
10	17	G in GFTTTL	2.56	**G 2.56 (INCL disease causing)**
10	18	F in FTTTLY	2.57	**1 after G 2.56 (INCL disease causing)**
10	18	G in GGFTTT	2.55	**1 before G 2.56 (INCL disease causing)**
10	9	F in FMVFGG	2.52	no match
11	18	K in KLRTPL	12.49 (ICL 1)	no match
11	14	T in TTTLYT	2.56	**2 after G 2.56 (INCL disease causing)**
11	18	L in LNYILL	2.39	no match
11	16	I in ILLNLA	2.42	no match
11	17	A in AIERYV	2.47	no match
12	18	N in NYILLN	2.40	no match
12	18	L in LYVTVQ	1.54	no match
12	13	L in LLNLAV	2.43	no match
12	18	N in NYILLN	2.40	no match
13	18	A in AEPWQF	1.27	no match
13	15	I in IMLGFP	1.43	no match
13	11	T in TLGGEI	3.33	no match
13	11	V in VCKPMS	3.54	no match
